# Anaesthesia for serial whole-lung lavage in a patient with severe pulmonary alveolar proteinosis: a case report

**DOI:** 10.1186/1752-1947-2-360

**Published:** 2008-11-27

**Authors:** Stephen T Webb, Adrian JR Evans, A James Varley, Andrew A Klein

**Affiliations:** 1 Department of Anaesthesia & Intensive Care, Papworth Hospital, Cambridge CB23 3RE, UK

## Abstract

**Introduction:**

Pulmonary alveolar proteinosis is a rare condition that requires treatment by whole-lung lavage. We report a case of severe pulmonary alveolar proteinosis and discuss a safe and effective strategy for the anaesthetic management of patients undergoing this complex procedure.

**Case presentation:**

A 34-year-old Caucasian man was diagnosed with severe pulmonary alveolar proteinosis. He developed severe respiratory failure and subsequently underwent serial whole-lung lavage. Our anaesthetic technique included the use of pre-oxygenation, complete lung separation with a left-sided double-lumen endotracheal tube, one-lung ventilation with positive end-expiratory pressure, appropriate ventilatory monitoring, cautious use of positional manoeuvres and single-lumen endotracheal tube exchange for short-term postoperative ventilation.

**Conclusion:**

Patients with pulmonary alveolar proteinosis may present with severe respiratory failure and require urgent whole-lung lavage. We have described a safe and effective strategy for anaesthesia for whole-lung lavage. We recommend our anaesthetic technique for patients undergoing this complex and uncommon procedure.

## Introduction

Pulmonary alveolar proteinosis (PAP) is a rare disorder characterised by the intra-alveolar accumulation of lipoproteinaceous material that is now thought to be surfactant [1]. The mainstay of treatment is whole-lung lavage (WLL), and we would like to present a case of this disease to illustrate a safe anaesthetic technique to facilitate this procedure.

## Case presentation

A 34-year-old Caucasian man presented to a hospital in the UK with a 1-month history of progressive exertional dyspnoea and non-productive cough. He was a current cigarette smoker but had no other medical problems. He was found to be severely hypoxaemic while breathing room air at rest (arterial haemoglobin oxygen saturation, SaO_2 _87%; arterial partial pressure of oxygen, PaO_2 _5.4 kPa) and chest X-ray showed bilateral patchy air-space infiltration. Pulmonary function testing demonstrated a restrictive ventilatory defect (forced expiratory volume in 1 s, FEV1 2.4 L; forced vital capacity, FVC 2.5 L; FEV1/FVC 43%) and impaired diffusion capacity (carbon monoxide diffusion capacity 45% of predicted value). Thoracic computed tomography indicated that the right lung was more severely diseased than the left. Broncho-alveolar lavage (BAL) fluid cytological examination was suggestive of PAP. He was admitted to a specialist cardiothoracic unit for urgent lung lavage.

On arrival in the operating room, the patient was dyspnoeic, cyanosed and severely hypoxaemic despite breathing high-flow oxygen via a facemask (SaO_2 _< 85%). Electrocardiographic and invasive arterial pressure monitoring were established. During pre-oxygenation, SaO_2 _improved to >90%. Anaesthesia was induced with propofol and fentanyl and subsequently maintained with propofol and remifentanil infusions. A non-depolarising neuromuscular blocking agent was administered to facilitate tracheal intubation. Oxygenation saturation remained stable following induction of anaesthesia. A 39 mm left-sided double-lumen endotracheal tube was inserted and its correct position confirmed by fibreoptic bronchoscopy. Airway pressure, tidal volume and end-tidal carbon dioxide concentration were continuously monitored as well as regular arterial blood gas analysis. WLL was performed with the patient in the supine position on the operating table. One-lung ventilation of the left lung was commenced just before initiation of lavage of the right lung. Under fibreoptic bronchoscopic control, a respiratory physician carried out repeated cycles of instillation of 1 L of 0.9% saline solution at body temperature followed by passive drainage under gravity. In order to achieve maximal filling and drainage of all lung segments, an experienced physiotherapist performed manual chest vibration and percussion. Various positional manoeuvres were also used to facilitate run-in and run-out of fluid. During fluid inflow and outflow, airway pressure and tidal volume were closely monitored to assess for leakage of fluid from the non-ventilated lung into the ventilated lung. Initially, milky fluid effluent was obtained and a total lavage volume of 10–15 L was necessary to obtain clear fluid effluent. The procedure lasted approximately 2 hours. At the end of the procedure, two-lung ventilation was commenced and recruitment manoeuvres were applied to restore expansion of both lungs. Satisfactory oxygenation was maintained throughout the procedure during both two-lung and one-lung ventilation (SaO_2 _> 90% and PaO_2 _> 8 kPa). Left-sided WLL was planned within the next 24 hours. Hence, the double-lumen endotracheal tube was exchanged for a 9.0 mm single-lumen tube and the patient was transferred to the intensive care unit (ICU) for ventilatory support. For left-sided WLL, the single-lumen endotracheal tube was exchanged for a 39 mm left-sided double-lumen tube and an identical technique for WLL was employed. The procedure was better tolerated with improved oxygenation compared to the previous WLL. At the end of the procedure, following an endotracheal tube exchange, the patient was transferred to ICU where he was extubated within 8 hours. Manual chest physiotherapy techniques and positioning manoeuvres were continued postoperatively.

Bilateral sequential WLL in the same session was performed on two occasions in the subsequent few weeks. Three further unilateral WLL procedures (one right-sided and two left-sided) were carried out in the following 6 months. A similar anaesthetic technique was used for each WLL procedure. Serial WLL resulted in clinical, physiological and radiological improvement for the patient and eventual remission of the disease.

## Discussion

Recent insights into the pathogenesis of PAP suggest that in the most common form, acquired (idiopathic) PAP, autoimmunity against pulmonary granulocyte-macrophage colony-stimulating factor (GM-CSF) plays a major role. Inhibition of GM-CSF results in impaired function of alveolar macrophages, disruption of surfactant homeostasis and reduced surfactant clearance from alveoli. Acquired PAP typically affects middle-aged men with a history of smoking who present with progressive exertional dyspnoea. Investigations reveal radiographic bilateral patchy air-space infiltration, restrictive pulmonary function, impaired diffusion capacity and milky broncho-alveolar lavage (BAL) fluid rich in alveolar macrophages.

Although increasing evidence indicates that GM-CSF therapy may be beneficial for patients with PAP, the mainstay of treatment is whole-lung lavage (WLL). The postulated therapeutic rationale of WLL is the washout of pathological alveolar material and removal of anti-GM-CSF autoantibodies. Although selective lobar lavage using local anaesthesia has been described, lung separation under general anaesthesia and lavage of the non-ventilated lung remain the standard treatment for PAP since first employed by Ramirez-Rivera [2]. Anaesthesia for WLL is undoubtedly hazardous: the use of one-lung ventilation for broncho-alveolar instillation and drainage of large volumes of fluid in the setting of pre-existing respiratory failure put the patient at risk of profound hypoxaemia and at risk of flooding of the ventilated lung. The pathophysiology of hypoxaemia is related to ventilation-perfusion mismatch during lung lavage: during the filling phase, perfusion of the non-ventilated lung is reduced by compression of the pulmonary vasculature and hence shunt is reduced; however, during the drainage phase, reperfusion of the non-ventilated lung increases shunt causing hypoxaemia.

Our experience suggests that good teamwork with the respiratory physician and the physiotherapist throughout this prolonged procedure is necessary for safe WLL. We used a left-sided double-lumen endotracheal tube for all procedures. We avoided the use of a right-sided tube, as it tends to block the orifice of the right upper lobe bronchus. Additionally, the shape of the cuff and the presence of the right upper lobe ventilation slit make an airtight seal difficult to achieve. The use of positive end-expiratory pressure (PEEP) applied to the ventilated lung may improve oxygenation during the filling phase, although during the drainage phase, it may augment the shunt through the non-ventilated lung. Monitoring of airway pressure and tidal volume during one-lung ventilation is crucial to detect fluid leakage into the ventilated lung. An increase in airway pressure or decrease in tidal volume may indicate a reduction in compliance of the ventilated lung and fluid leakage should be considered. Fibreoptic bronchoscopic inspection will confirm if flooding of the ventilated lung has occurred. Treatment involves rapid endobronchial suctioning followed by effective re-expansion of the flooded lung. Use of continuous breath-by-breath compliance monitoring may be a useful additional tool. Patient positioning should be carried out carefully in order to avoid endotracheal tube movement. The full lateral position with the lung undergoing lavage uppermost should be avoided if possible, as there is a significant risk of flooding of the dependent ventilated lung.

Various other strategies have been suggested for the management of hypoxaemia during WLL including manual ventilation of partially fluid-filled lung [3], intermittent double-lung ventilation [4], concomitant use of inhaled nitric oxide and almitrine [5], and pulmonary artery occlusion of the non-ventilated lung using a pulmonary artery catheter [6]. Hyperbaric oxygen and veno-venous extracorporeal membrane oxygenation have also been reported to be useful in patients undergoing WLL. The use of postoperative differential lung ventilation and extubation criteria based on restoration of pre-lavage lung compliance has been recommended [7].

We have described an acceptable anaesthetic technique for WLL in a patient with severe respiratory failure due to PAP. We advocate multidisciplinary team working, use of pre-oxygenation, complete lung separation with a left-sided double-lumen endotracheal tube, one-lung ventilation with PEEP, appropriate ventilatory monitoring, cautious use of positional manoeuvres and single-lumen endotracheal tube exchange for short-term postoperative ventilation.

## Competing interests

The authors declare that they have no competing interests.

## Authors' contributions

STW conceived the case report, drafted and revised the manuscript, and reviewed the relevant literature relating to this subject; AJRE drafted the manuscript; AJV drafted the manuscript; AAK revised the manuscript and gave approval for submission for publication.

## Consent

Written informed consent was obtained from the patient for publication of this case report and any accompanying images. A copy of the written consent is available for review by the Editor-in-Chief of this journal.
